# Spatially inhomogeneous inverse Faraday effect provides tunable nonthermal excitation of exchange dominated spin waves

**DOI:** 10.1515/nanoph-2023-0626

**Published:** 2024-01-19

**Authors:** Denis M. Krichevsky, Vladislav A. Ozerov, Alexandra V. Bel’kova, Daria A. Sylgacheva, Andrey N. Kalish, Svetlana A. Evstigneeva, Alexander S. Pakhomov, Tatiana V. Mikhailova, Sergey D. Lyashko, Alexander L. Kudryashov, Evgeny Yu. Semuk, Alexander I. Chernov, Vladimir N. Berzhansky, Vladimir I. Belotelov

**Affiliations:** Russian Quantum Center, 143025, Skolkovo, Moscow Region, Russia; Moscow Institute of Physics and Technology (National Research University), 141700, Dolgoprudny, Russia; V.I. Vernadsky Crimean Federal University, 295007, Simferopol, Russia; Photonic and Quantum Technologies School, Faculty of Physics, Lomonosov Moscow State University, 119991, Moscow, Russia; New Spintronic Technologies, 121205, Moscow, Russia

**Keywords:** the inverse Faraday effect, magnetophotonic crystal, iron garnet, spin waves

## Abstract

We demonstrate optical nonthermal excitation of exchange dominated spin waves of different orders in a magnetophotonic crystal. The magnetophotonic structure consists of a thin magnetic film and a Bragg stack of nonmagnetic layers to provide a proper nonuniform interference pattern of the inverse Faraday effect induced by light in the magnetic layer. We found a phenomenon of the pronounced phase slippage of the inverse Faraday effect distribution when the pump wavelength is within the photonic band gap of the structure. It allows to tune the interference pattern by a slight variation of light wavelength which results in the modification of excitation efficiency of the different order spin waves. The approach can be applied for different magnetic dielectrics expanding their application horizons for spin-wave based devices.

## 1 Introduction

Efficient interaction between light and a magnet is vital for breakthrough technologies, such as magnetophotonics [1], [2], [3], [4], [5], [6], [7], all-optical energy-efficient magnetic recording [8] and spin-wave manipulation [9], [10], [11]. The latter one is an intrinsic part for spin-waves computation [12], [13] in which high frequencies are of a great demand.

Precession of magnetic moments and propagation of these moments in the form of spin waves in magnetically ordered materials were first predicted almost a century ago by Bloch [14] and only a decade ago it was demonstrated that spatially shaped light can be used for the optical excitation of spin waves with the directional control [9]. The advances in ultrafast optical manipulation of magnetic order [8], [15], [16] provided new ways for the non-thermal excitation of propagating magnetostatic spin waves [9], [17] in contrast to conventional microstrip antenna or coplanar waveguide [13], [18], [19], leading to the tunable energy-efficient spin wave manipulation [9], [20] and even reconfigurable optomagnonic logic gates [21].

One way to optically and non-thermally excite and control spin waves is based on the inverse magnetooptical effects [22], in particular, the inverse Faraday effect (IFE). The effect occurs when a circularly polarized light pulse, passing through a magnetic medium, influences spins due to the stimulated Raman scattering, which is described in terms of an effective magnetic field induced by light. Experimentally, this method is implemented with the use of femtosecond laser pulses and it provides a substantial tunability of the excited spin waves properties in terms of their type [17], wavelength [23] and initial phase [24]. However, the most of the excited spin waves are described by magnetic dipolar interaction and are magnetostatic spin waves of 0th order. In external magnetic fields of a moderate value their frequency is around few GHz. Launching the higher frequency spin waves which are urgently awaited by magnonics is still scarcely achievable.

Wavelengths of high-frequency exchange-dominated spin waves are submicron. Therefore, a key point to efficiently excite them is to make a kind of nonuniformity of either internal magnetic field or an excitation stimulus inside a sample. The former approach was successfully applied for launching spin waves conventionally by microwaves in a periodically structured ferromagnet [25], [26]. However, it is not possible to keep dealing with the microwave stimulus for uniform samples since microwave wavelengths far exceed their micron and especially nanometer thickness. One possibility is to use exchange torques [25], [26], however it requires metallic parts of the structure and inevitably generates excess heating due to large amplitudes of the control electric currents.

Optical means are potentially very promising in this respect since wavelength of visible light in a magnet is comparable to the magnet thickness. A most straightforward way to achieve nonuniform impact of light on spins is due to the optical absorption which provides the exponential attenuation of the light intensity across the film thickness. It results to a nonuniform spin excitation profile due to modification of the magnetic anisotropy [27] or some other thermal effects [20]. Such method has been proven quite efficient and allowed to excite, for example, short exchange spin waves with supersonic velocities [20]. Nevertheless, such approach still requires light absorption and, therefore, has an inevitable side effect of undesirable heating and thermal losses.

The next step forward would be to avoid thermal mechanism of the optical excitation of exchange spin waves. Necessary nonuniform profile of the optical field in a magnet can be established not by the absorption of light but rather due to the optical interference or optical modes inside the sample. The latter has been implemented in an iron garnet film periodically perforated with nanotranches which allowed to excite nonthermally exchange spin waves of first two orders and switch between them by variation of angle of the linear polarized pump pulse [28]. In that experiment the nanotranches played a two-sided role: they provided an inhomogeneous distribution of both the optical energy and internal magnetic field across the sample. The latter complicates spin wave profile by evoking harmonics with lateral spatial distribution of spin dynamics.

Here we demonstrate nonthermal optical excitation of exchange-dominated standing spin waves (SSW) in a magnetophotonic structure (MPhS) consisting of a smooth magnetic nanofilm covered with a dielectric nonmagnetic Bragg mirror. A periodic across film thickness spin excitation profile is achieved due to the interference of the incident laser pulse. Incident optical pump pulses are circularly polarized and therefore carry spin angular momentum and induce IFE effective magnetic field. Periodic distribution of this field with a period of 125–166 nm efficiently excites the exchange spin waves whose wavelength equals to the period of the optical pattern. By tuning excitation wavelength, we were able to adjust the IFE effective magnetic field distribution inside the magnetic layer and launch 3rd and 4th order exchange spin waves.

## 2 Results and discussion

We performed the experiments on the optical excitation of SSW using 200 fs laser pulses whose wavelength is swept in a relatively short rage between *λ*_*pm*_ = 610 nm and *λ*_*pm*_ = 685 nm. Pump and probe pulses were generated by Newport Mai Tai Ti:sapphire laser (80.68 MHz repetition rate) combined with Spectra-Physics Inspire Auto 100 parametric oscillator. A delayed pump pulse was modulated using a photoelastic modulator (Hinds instruments PEM 100). The probe pulse (820 nm) was linearly polarized by a Glan–Taylor prism. Polarization changes of the probe pulse due to the Faraday effects were measured using auto-balanced optical receiver in the lock-in detection scheme. The sample was placed in the in-plane field of an electromagnet.

**Figure 1:**
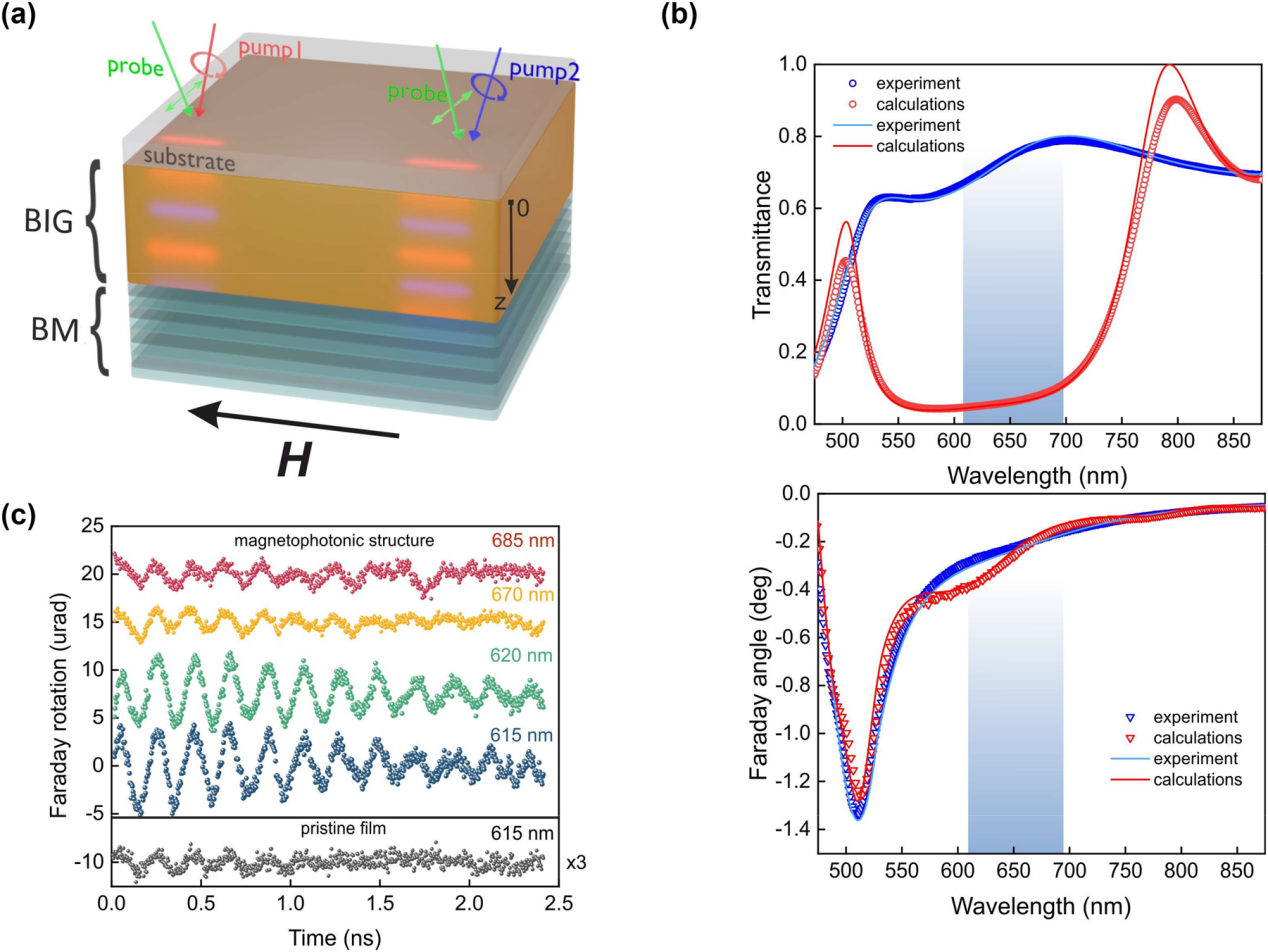
The MPhS and optically excited spin dynamics. (a) – schematics of the MPhS, consisting of a 250-nm thick bismuth iron-garnet layer sandwiched between SiO_2_/TiO_2_ Bragg mirror and GGG substrate. Bright maxima inside the BIG layer represent distribution of the IFE effective magnetic field induced by pumping at two different wavelengths: pump-1 (615 nm) and pump-2 (685 nm). (b) – Transmission (upper part) and Faraday rotation (lower part) spectra of the magnetophotonic crystal (red) and pristine 250-nm BIG film (blue). The blue shaded area indicates range of the pump wavelengths. (c) – The spin precession launched by pump pulses of different wavelengths (615 nm, 625 nm, 670 nm and 685 nm) and observed by the Faraday rotation of the probe pulses at 970 Oe magnetic field for the magnetophotonic structure and the pristine BIG film.

The considered MPhS consists of a 250 nm thick bismuth substituted iron garnet (BiLu)_3_Fe_5_O_12_ (BIG) film grown on a Gd_3_Ga_5_O_12_ paramagnetic substrate and covered by a dielectric SiO_2_/TiO_2_ (105/66 nm thick, respectively) Bragg mirror (Figure 1(a)). It is placed in an external magnetic field which fully saturates the magnetic film in plane. Wavelength of the circularly polarized pump pulses is adjusted within the photonic band gap of the MPhS where the reflection coefficient is high (Figure 1(b)). As a result, an interference pattern of the electromagnetic field of the laser pulse is formed. Since the pulse is circularly polarized it carries spin angular momentum and in accordance to the inverse Faraday effect induces the IFE effective magnetic field **H**_IFE_: $H_{\mathrm{IFE}}=-\frac{ig}{16\pi M_{s}}\left[E\times E^{*}\right]$ [22], where *g* is gyration coefficient of the magnetic film, *M*_*s*_ is its saturation magnetization, and **E**, E* are pump electric field vector and its complex conjugate. Due to the interference **H**_IFE_ becomes nonuniform across the film thickness and one should expect the optical pumping to excite standing spin waves whose wavelength and frequency are controlled by the pump wavelength.

**Figure 2:**
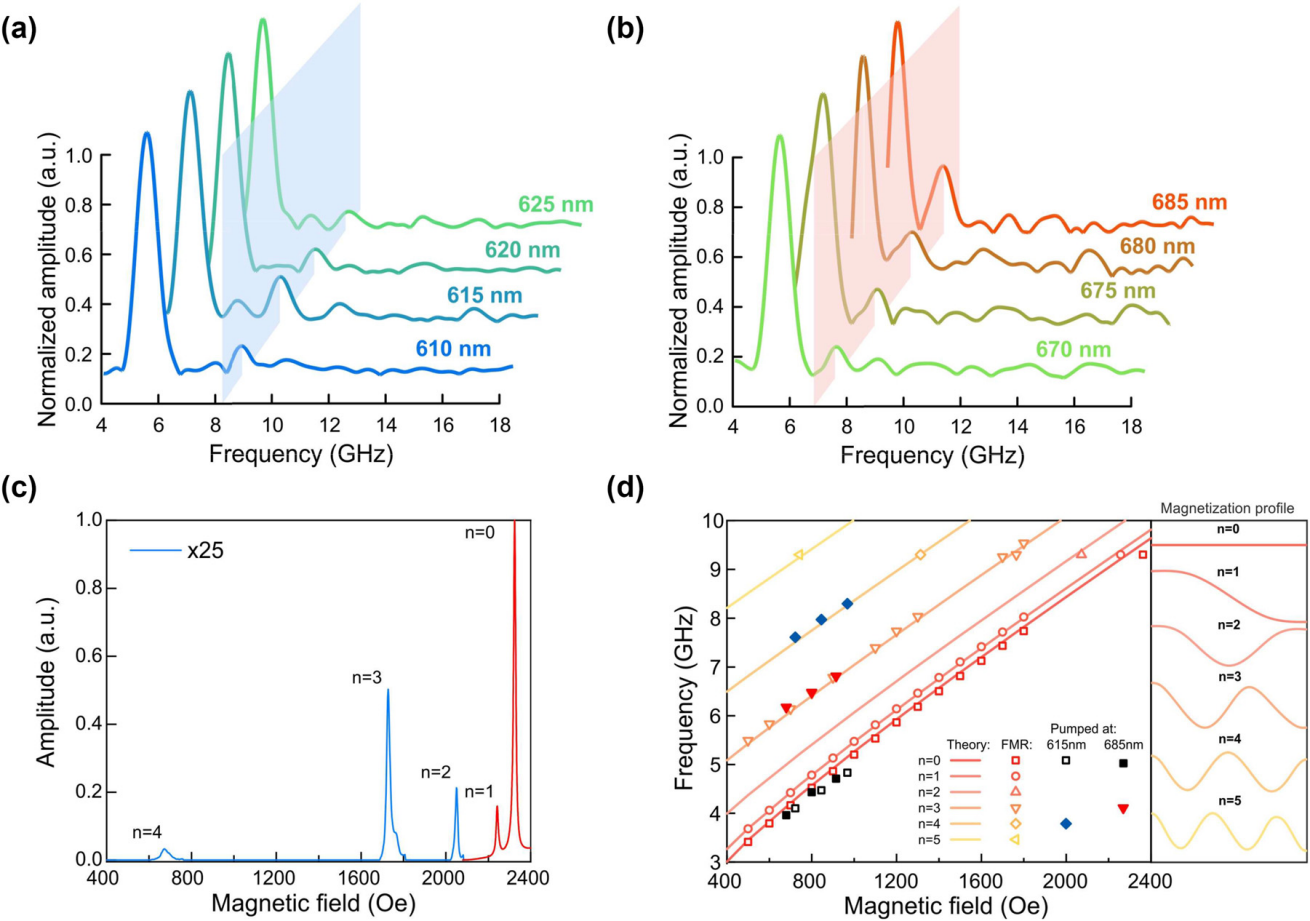
Spectral properties of the optically excited spin oscillations and comparison to the microwave excitation (FMR measurements). (a, b) – FFT spectra of spin oscillations pumped at various wavelengths at 970 Oe external magnetic field. (c) – FMR spectra of the sample at excitation frequency of 9.4 GHz. (d) – Dependance of the frequencies of different spin wave modes of the magnetic film excited by optical pumping (shaded symbols) and FMR (open symbols) on the external magnetic field. Solid curves represent corresponding calculated dependences (Supplementary Document, S1). Inset: Schematic mode profiles of considered modes.

The observed signals excited by different pumping wavelengths (Figure 1(c)) have different spectral composition as follows from their Fourier spectra (Figure 2(a and b)). For the pump wavelength of 610 nm the spectrum contains one pronounced peak at 4.8 GHz and a minor peak at 8.2 GHz. When the pump wavelength is increased a bit, the primary peak remains the same while the second peak gets larger at *λ*_*pm*_ = 615 nm and then decreases. Therefore, pumping at *λ*_*pm*_ = 615 nm clearly provides two different spin modes: the low frequency one with large amplitude and the high frequency one with a smaller amplitude. Similar behavior takes place for the wavelength ranging from *λ*_*pm*_ = 670 nm to *λ*_*pm*_ = 685 nm: the main peak keeps at 4.8 GHz, while the subsidiary one increases for wavelength grows and maximizes at 685 nm. It should be noted that for this wavelength window the second peak appears at a frequency of 6.8 GHz which is smaller than for the secondary peak at the previous pumping wavelength range. Herein, the peak at 8.2 GHz disappears.

**Figure 3:**
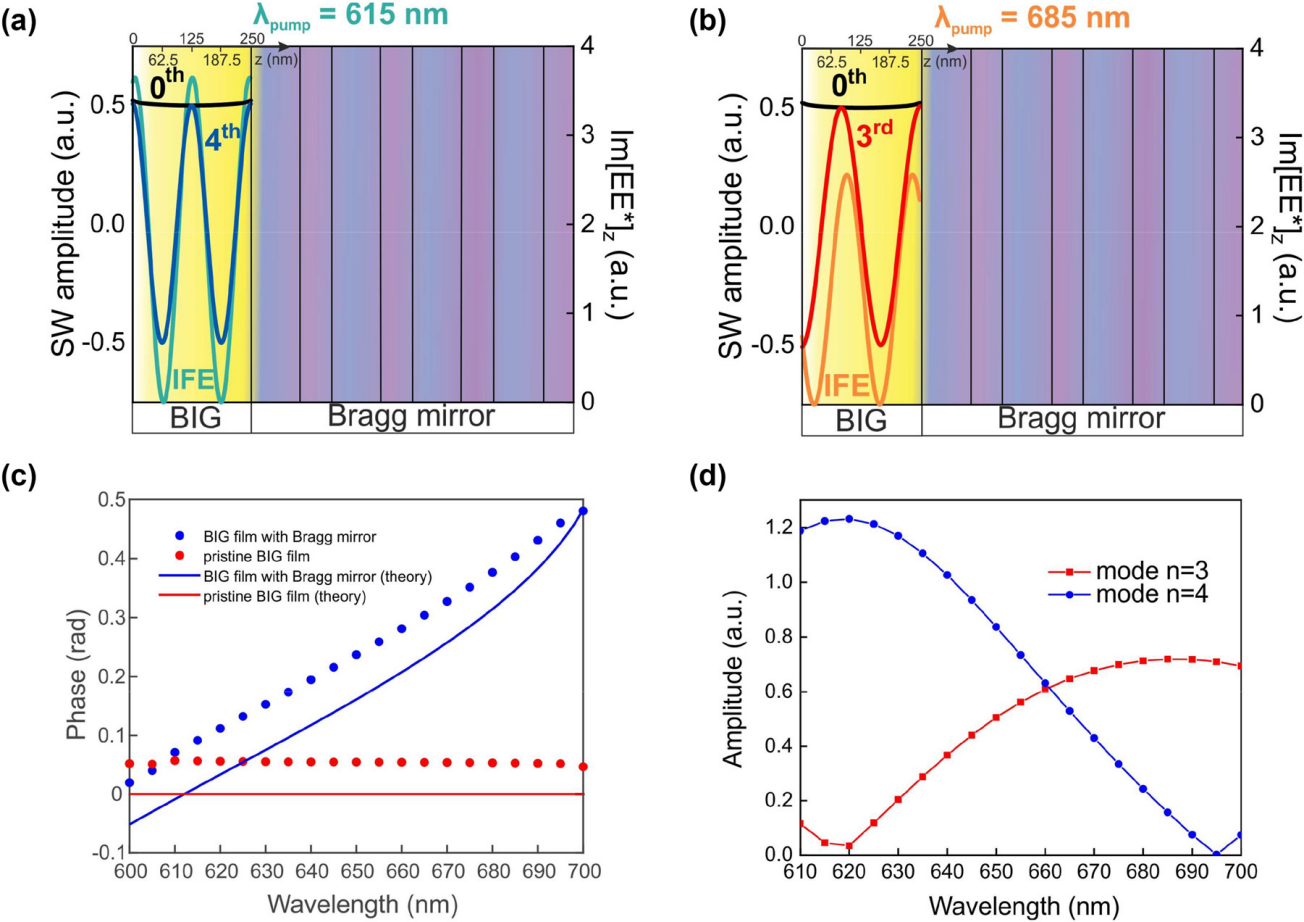
Profiles of the SSWs. (a, b) – numerically calculated IFE field distribution inside the structure at 615 and 685 nm and profiles of the SSW modes of 0th, 3rd and 4th order. (c) – The phase of the IFE field inside the BIG layer as a function of pump wavelength for pristine (red color) and photonic crystal (blue color) covered films. Dots show the phase deduced from the simulated field distribution, lines give the calculations by Eq. (4) (see Supplementary Document, S2 for details). (d) – Numerically calculated dependence of the third and fourth standing spin wave modes amplitude as a function of wavelength. Magnetic field is 980 Oe.

To shed a light on the observed oscillations origin and behavior we measured ferromagnetic resonance (FMR) in the sample. The investigation of magnetic properties of the MPhS and the possibility of excitation of standing spin waves in the structure was carried out on a SPINSCAN EPR spectrometer at a fixed frequency of 9.4 GHz (Figure 2(c)). In frequency-sweep VNA-FMR experiment the microwave absorption by the sample was measured at fixed external applied in-plane magnetic fields while driving the frequency. The frequency range of the sweep was from 2 to 8 GHz and the external magnetic field was varied from 900 to 2000 Oe. Figure 3(d) combines data from the magnetooptical pump-probe experiment, theoretical calculations, fixed and sweep frequency FMR experiments.

Generally, since microwaves are uniformly distributed along the submicron thickness of the magnetic film it is very difficult to excite SSWs by them. However, in our FMR experiment the sample was placed in the microwave cavity which allowed us to excite and detect SSWs of several orders though with very small amplitudes (Figure 2(c)). Ratio of the amplitudes of the 0th-1st-2nd-3rd-4th modes are 1:6:50:119:757, which confirms ultra-low excitation efficiency by microwave means. Figure 2(d) compares spectral positions of the detected FMR resonances (open symbols) and corresponding frequencies of the modes observed in the pump-probe experiment (shaded symbols, pump wavelength was set to 615 nm and 685 nm). Solid curves in Figure 2(d) represent dependence of the calculated frequencies of SSW modes of different orders (solid curves) on magnetic field (Supplementary Document, Section S1). It is seen that frequency of the low-frequency optically excited mode (grey and black shaded symbols) closely follows the frequency of the main FMR peak (open red symbols) and nicely corresponds to the theoretical curve for the uniform spin dynamics in the film (*n* = 0). Furthermore, the frequency of the high-frequency mode excited at 685 nm almost coincides with the fourth FMR peak and is in a good agreement with the calculated curve for the 3rd order SSW (with a wavelength of ∼125 nm). Similarly, the high-frequency mode excited at 615 nm is attributed to SSW of the 4th order (with a wavelength of ∼166 nm). Consequently, one can firmly conclude that, indeed, the modes observed in the pump-probe experiment are higher order exchanged spin waves. To study this phenomenon in detail let’s consider the distribution of the IFE effective field inside the magnetic layer and its impact on the spin modes excitation.

Since the external magnetic field is saturating and is applied in-plane the *n*th order SSW can be characterized by the out-of-plane dynamic magnetization component *M*_*nz*_(*z*, *t*). The SSW mode profile is given by $m_{n}\left(z\right)$ – the normalized amplitude of *M*_*nz*_(*z*, *t*). The excitation efficiency of the SSW modes is directly connected with an overlap between the IFE effective magnetic field distribution H_IFE_(*z*) and *n*th order SSW mode profile [29], [30], [31] (Supplementary Document S3):

(1) $$S_{\text{pm}}=\int_{0}^{d}H_{\text{IFE}}\left(z\right)m_{n}\left(z\right)dz/\left|\int_{0}^{d}H_{\text{IFE}}\left(z\right)dz\right|,$$

where *d* is the film thickness. Consequently, to maximize *S*_pm_ it is crucial to adjust H_IFE_(*z*) to $m_{n}\left(z\right)$ .

As light is incident close to the sample normal the optical interference forms a periodic pattern of the out-of-plain component of H_IFE_(*z*) across the film thickness (Supplementary Document S2):

(2) $$\begin{array}{ll} H_{\mathrm{IFE}}\left(z\right)\propto g\left[A_{0}\left(\lambda_{pm}\right)\right. & +\left.A\left(\lambda_{pm}\right)\cos^{2}\left(k\left(\lambda_{pm}\right)\left(z-d\right)\right.\right.\\ & +\left.\left.\varphi \left(\lambda_{pm}\right)\right)\right], \end{array}$$

where $A_{0}\left(\lambda_{pm}\right)$ , $A\left(\lambda_{pm}\right)$ and *φ*(*λ*_*pm*_) are parameters of the optical intensity distribution inside the BIG layer, *n*_2_ is the refractive index of the magnetic layer, *k*(*λ*_*pm*_) = 2*πn*_2_/*λ*_*pm*_, *d* is thickness of the BIG layer, and *z* is the coordinate normal to the film surface, *z* = 0 is at the interface between BIG layer and GGG substrate. The essential feature of the Bragg reflector cover of the magnetic film is that for the photonic bandgaps $A_{0}\left(\lambda_{pm}\right)=0$ , so that

(3) $$H_{\mathrm{IFE}}\left(z\right)\propto gA\left(\lambda_{pm}\right)\cos^{2}\left(k\left(\lambda_{pm}\right)\left(z-d\right)+\varphi \left(\lambda_{pm}\right)\right),$$

where

(4) $$\begin{array}{c} \varphi \left(\lambda_{pm}\right)=\text{atan}\left(-in_{3}/n_{2}\right), \end{array}$$

*n*_3_ is the complex effective refractive index of the Bragg mirror. Eq. (3) is illustrated by light blue and orange curves in Figure 3(a and b).

As it is seen from Eq. (3), both period and phase of $H_{\mathrm{IFE}}\left(z\right)$ are sensitive to *λ*_*pm*_. While the period of $H_{\mathrm{IFE}}\left(z\right)$ is inversely proportional to *λ*_*pm*_, the phase is almost linear in *λ*_*pm*_ (Figure 3(c)). This feature is unique for the Bragg reflector cover as is illustrated in Figure 3(c). Figure 3(c) shows the phase $\varphi \left(\lambda_{pm}\right)$ deduced from the simulated optical field distributions for the case of the Bragg mirror (blue dots) and for the case when the Bragg mirror is replaced by air layer (red dots). The direct calculation of $\varphi \left(\lambda_{pm}\right)$ by Eq. (4) is also shown (solid lines) (see Supplementary Document S2 for details). This effect of strong phase slippage $\varphi \left(\lambda_{pm}\right)$ of the IFE appears in the MPhS for the wavelengths inside the photonic band gap and is crucial for SSW control as will be discussed below.

As for $m_{n}\left(z\right)$ distribution, the 0th order SSW mode is quasiuniform across the magnetic film ( $m_{0}\left(z\right)\approx \text{const}$ ), whereas higher order modes have profiles close to sine or cosine functions (blue and red curves in Figure 3(a and b)). Having uniform distribution, the 0th-mode is excited by both uniform and nonuniform IFE fields since the pumping efficiency *S*_pm_ remains relatively large for these cases. On the contrary, as it follows from Eq. (1), the higher spin modes require periodic H_IFE_(*z*) with the period comparable to their wavelength. The excitation efficiency (namely, modulus of the amplitude) of the 3rd and 4th order SSWs as a function of pump wavelength is summarized in Figure 3(b). The 4th order SSWs is excited most efficiently at 615–620 nm, where its profile almost coincides to the profile of H_IFE_(*z*) (blue curve and symbols in Figure 3(d)). Increase of the pump wavelength detunes H_IFE_(*z*) profile from the 4th-SSW one and amplitude of 4th-SSW drops down almost to zero at *λ*_*pm*_ = 695 nm. At the same time, H_IFE_(*z*) profile gets closer to the 3rd-SSW one and amplitude of 3rd-SSW grows (red curve and symbols in Figure 3(d)). It reaches parity with 4th-SSW at *λ*_*pm*_ = 660 nm and grows further up to *λ*_*pm*_ = 685 nm where it maximizes. Therefore, changing pump wavelength, one could switch between different orders of exchange spin waves or excite their superposition.

Tuning of H_IFE_(*z*) between optimum for excitation of 4th-SSW and 3rd-SSW is possible due to dependence of its spatial period and, most importantly, phase on the pump wavelength: *k*(*λ*_*pm*_) and *φ*(*λ*_*pm*_) (see Eq. (2)). It is important to note the role of the Bragg reflector. Indeed, if the Bragg reflector is replaced by a dielectric layer, then *φ*(*λ*_*pm*_) would be constant and to tune between 4th-SSW and 3rd-SSW one would need to change $k\left(\lambda_{pm}\right)$ substantially, by Δ*k* = *π*/2*d*, which would require Δ*λ*_*pm*_ = d*n*_2_/3 = 207 nm. Here the presence of the Bragg reflector provides *φ*(*λ*_*pm*_) and one has to increase the pump wavelength by only Δ*λ*_*pm*_ = d*n*_2_/3 = 75 nm to get the same level of tunability.

**Figure 4:**
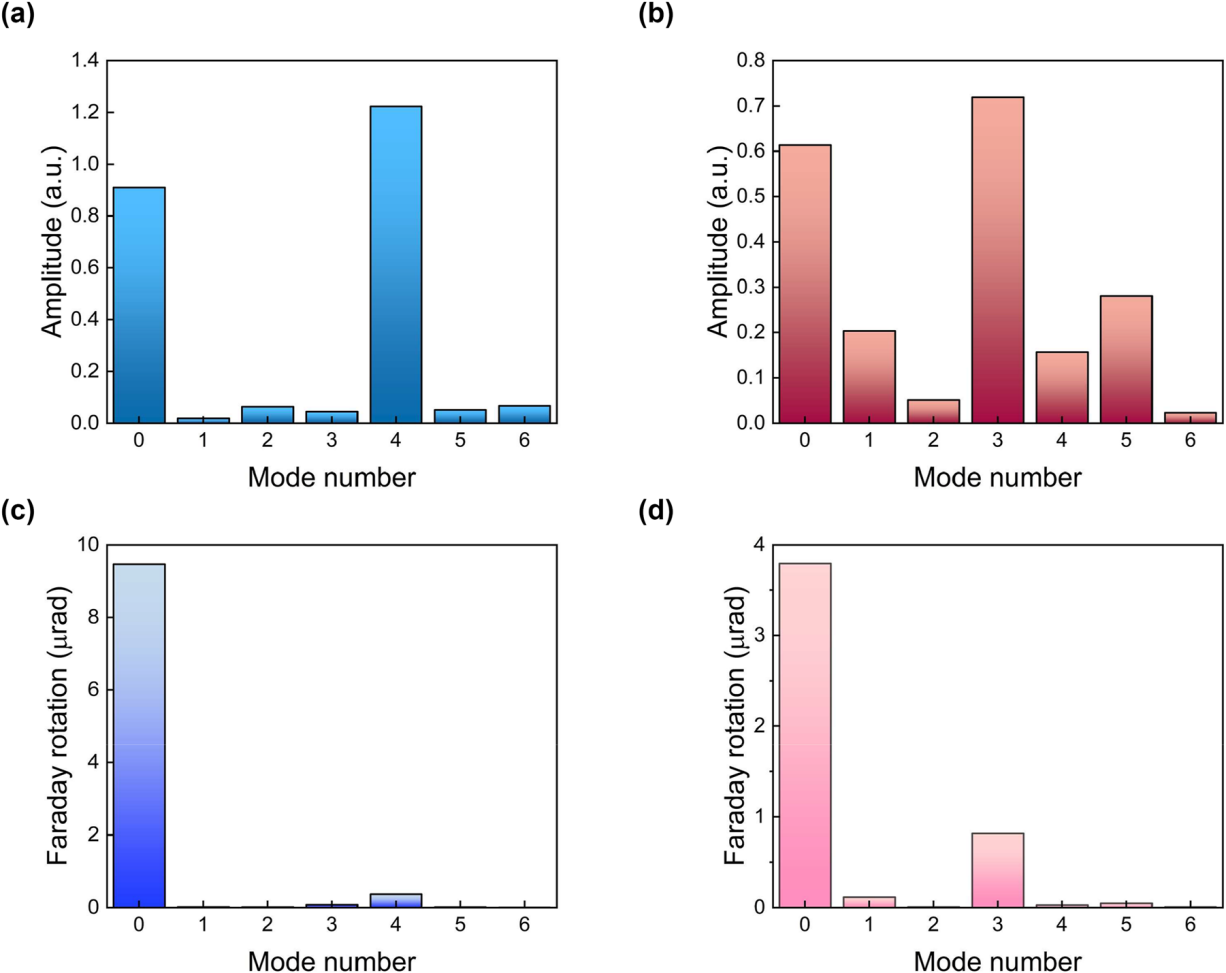
Amplitudes of the standing spin wave modes pumped at 615 (a, c) and 685 (b, d) nm. Panels (a) and (b) represent calculated amplitudes of the spin-wave modes without taking into account the detection process. Panels (c) and (d) represent calculated amplitudes of the spin-wave modes by taking into account the detection process. Magnetic field is 980 Oe.

Let’s now discuss relative values of the SSW mode amplitudes observed in the experiment. It is important to note that the magneto-optically probed SSW amplitudes might be significantly different from the SSW amplitudes in reality. The Faraday effect, which is widely employed for probing in the ultrafast experiments [22], [32], is sensitive to the dynamic component of magnetization which is along wavevector of the probe. If the probe pulse shines the sample at normal or close to normal incidence then the Faraday effect is proportional to the out-of-plane component of magnetization. In case of an SSW mode *m*_*n*_(*z*) and the probe pulse intensity *W*_pr_(*z*) are nonuniformly distributed across the film thickness. Consequently, the Faraday angle is proportional to the maximal value of the out-of-plane magnetization of *n*th mode, *M*_*nz*_, and to the probing efficiency $S_{\text{pr}}=\int_{0}^{h}W_{\text{pr}}\left(z\right)m_{n}\left(z\right)dz/\left|\int_{0}^{h}W_{\text{pr}}\left(z\right)dz\right|$ . For the case of 0th order mode typically observed in thin films, magnetization distribution remains quasi-homogeneous ( $m_{0}\left(z\right)\approx \text{const}$ ). Similarly, distribution of the probe in a magnetic film is also close to the uniform one (*W*_pr_(*z*) ≈ const), which makes detection efficiency of this mode quite large (*S*_pr_ ≈ 1). In the case of the MPhS pump and probe become non-uniform across the film and, as we discussed above, the non-uniform pumping launches higher order modes (see Eq. (2)) whose amplitude is also thickness dependent (*m*_*n*_(*z*)). As a result, the Faraday signal of the probe beam is determined not only by a value of *M*_*nz*_ but also by relative distribution of *W*_pr_(*z*) and *m*_*n*_(*z*). In some cases, the observed Faraday effect could be quite small even for large *M*_*nz*_. Following these ideas, we numerically calculated (Supplementary Document S1) the amplitudes of the SSW modes excited by the pump pulse without taking into account magnetooptical detection (Figure 4(a,b)) and the amplitudes of the SSW modes detected by the Faraday rotation of 820 nm probe pulses (Figure 4(c,d)).

A 615 nm pump pulse excites 0th and 4th order SSW modes with similar amplitudes. Moreover, the amplitude of the 4th-mode is even a bit larger (see Figure 4(a)). However, after detection relative amplitudes of the 0th and 4th modes are quite different, their ratio is around 20, since for the 0th-mode the detection integral *S*_pr_ is much larger (Figure 4(c)). A similar situation is noticed for the 685 nm pump which excites mostly 0th and 3rd SSW modes: the ratio of the mode amplitudes after detection is 1:5 (Figure 4(d)) while without taking into account detection process amplitudes are almost the same (Figure 4(b)). The differences in mode amplitudes between experimental (Figure 1(c)) and numerically calculated (Figure 4(c and d)) values are primarily due to incident angle variation for both pump and probe beams, as well as structure inhomogeneities caused by the fabrication process. It should be mentioned that for excitation of low-order standing spin wave modes, such as the 1st or the 2nd one, the BIG layer thickness can be decreased to 125 nm. This will result in optical defect mode of the MPhS to occur at ∼525 nm while the photonic band gap remains unchanged (see Supplementary Document, Figure S1a). For this case the spatial distribution of the effective magnetic field of the IFE will coincide with the 1st, the 2nd or the 3rd standing spin wave mode (see Supplementary Document, Figure S1b).

## 3 Conclusions

Our experiments demonstrate spectrally adjustable excitation of high-order SSW caused by inhomogeneous effective magnetic field formed inside the magnetic layer of the MPhS by femtosecond laser pulses. The MPhS provides necessary distribution of the inverse Faraday effect magnetic field and also gives a high level of tunability since spatial phase of the inverse Faraday effect inside its magnetic layer becomes strongly dependent on the pump wavelength. This effect appears in the photonic band gap of the structure. As a result, 3rd and 4th order spin modes are shown to be induced with an efficiency close to the 0th mode efficiency which is not reachable by microwave means. To tune between these modes variation of the pump wavelength by only 75 nm is enough. However, visibility of the spin modes in the experiment is several times lower due to not optimal distribution of the probe pulse across the film thickness. To increase the probing efficiency the probe wavelength should be chosen to get distribution of the probe beam close the pump one. Optical excitation of high-order exchange spin modes is of prime importance for data processing applications involving optospintronic and optomagnonic devices with higher-frequencies demands.

## Supplementary Material

Supplementary Material Details
