# Supplementary material for: Spatially inhomogeneous inverse Faraday effect provides tunable nonthermal excitation of exchange dominated spin waves
Source: Nanophotonics. 2024 Jan 19;13(3):299–306. doi: 10.1515/nanoph-2023-0626 (PMC11502009; doi:10.1515/nanoph-2023-0626)
Supplement: Supplementary file 1 — Supplementary Material Details [file j_nanoph-2023-0626_suppl_001.docx]

**Supplemental Document**

Spatially inhomogeneous inverse Faraday effect provides tunable nonthermal excitation of exchange dominated spin waves

Krichevsky D.M.^1,2^, Ozerov V.A.^1,2^, Bel’kova A.V.^1,4^, Sylgacheva D.A.^1^, Kalish A.N.^1,4^, Evstigneeva S.A.^1^, Pakhomov A.S.^1,2,5^, Mikhailova T.V.^3^, Lyashko S.D.^3^, Kudryashov A.L.^3^, Semuk E.Yu.^3^, Chernov A.I.^1,2^, Berzhansky V.N.^3^, Belotelov V.I.^1,3,4^

^1^Russian Quantum Center, 143025, Skolkovo, Moscow Region, Russia

^2^Moscow Institute of Physics and Technology (National Research University), 141700, Dolgoprudny, Russia

^3^ V.I. Vernadsky Crimean Federal University, 295007, Simferopol, Russia

^4^Photonic and Quantum Technologies School, Faculty of Physics, Lomonosov Moscow State University, 119991, Moscow, Russia

^5^New Spintronic Technologies, 121205, Moscow, Russia

**S1. Landau-Lifshitz-Gilbert equation and SSW.**

Magnetization dynamics launched by ultrashort laser pulses can be investigated on the basis of the Landau-Lifshitz-Gilbert equation:

$$\frac{d\mathbf{M}}{dt}=-\gamma\left[ \mathbf{M}\times\mathbf{H}_{eff} \right]+\frac{\alpha}{\left| \mathbf{M} \right|}\left[ \mathbf{M}\times\frac{d\mathbf{M}}{dt} \right]. (S1.1)$$

Here the effective magnetic field acting on magnetization is $\mathbf{H}_{eff}=\mathbf{H}+4\pi\mathbf{M}-\frac{2K_{U}}{M}\mathbf{e}_{z}+A\Delta\mathbf{M}+\mathbf{H}_{\mathrm{IFE}}$, where$K_{U}$ is uniaxial anisotropy constant, $A$ is exchange constant, $\gamma$ is gyromagnetic ratio and $\alpha$ is Gilbert damping constant considered to be small ($\alpha\sim{10}^{-3}$). The direction of the external magnetic field $\mathbf{H}\boldsymbol{=}\mathbf{e}_{x}H$ corresponds to the x-axis (which lies in a sample plane), while the IFE-field $\mathbf{H}_{\mathrm{IFE}}=\mathbf{e}_{z}f(t)h(z)$ is oriented along z-axis (which is perpendicular to the sample plane). We can define the spherical coordinate system in terms of $\theta,\varphi$ angles, where $\theta$ is a deflection angle between the magnetization $\mathbf{M}$ and its in-plane projection $\mathbf{M}_{\mathrm{xy}}$, whereas $\varphi$ is a deflection angle between the in-plane projection $\mathbf{M}_{\mathrm{xy}}$ and the external field $\mathbf{H}$. In this coordinate system we have $M_{y}=\left| \mathbf{M} \right|\cos\theta\sin\varphi; M_{z}=\left| \mathbf{M} \right|\sin\theta$ and Eq. (S1.1) is rewritten as:

$$\left\{ \begin{aligned} \dot{\theta}=\alpha\dot{\varphi}+\gamma H\varphi-\gamma AM\varphi^{''}, \\ \dot{\varphi}=-\alpha\dot{\theta}-\gamma\tilde{H}\theta+\gamma AM\theta^{''}+\gamma f(t)h(z). \end{aligned} \right. (S1.2)$$

Here we assumed that the precession angle is small ($\theta\ll1$, $\varphi\ll1$), and the derivatives are denoted by $\theta^{''}=\frac{\partial^{2}\theta}{\partial z^{2}} , \varphi^{''}=\frac{\partial^{2}\varphi}{\partial z^{2}} , \dot{\theta}=\frac{\partial\theta}{\partial t} ,\dot{\varphi}=\frac{\partial\varphi}{\partial t}$ . For convenience, we introduced the notation $\tilde{H}=H+4\pi M-\frac{2K_{U}}{M}$, where $H=\left| \mathbf{H} \right|, M=|\mathbf{M}|$ .

The term $\gamma f\left( t \right)h(z)$ in Eq. (S1.2) (here $h(z)$ corresponds to the average value of the magnetic field of the inverse Faraday effect during the pulse propagation) is non-zero only during the small time of laser pulse propagation $\Delta t$, meaning that it is responsible only for the establishing of the initial conditions for $\theta,\varphi$. If we integrate Eq. (S1.2) by $t$ from 0 to $\Delta t$ using $\int_{0}^{\Delta t} f(t)dt=\Delta t$, we will find the initial conditions for $\theta,\varphi$ after the instant stimulus of a laser pump:

$$\left\{ \begin{aligned} \varphi\left( z,t=\Delta t \right)=\gamma\Delta t\cdot h\left( z \right), \\ \theta\left( z,t=\Delta t \right)\cong0. \end{aligned} \right. (S1.3)$$

Besides, boundary conditions should also be taken into consideration. As shown in  [1], for the given configuration the boundary conditions take the form:

$$\left\{ \begin{aligned} \theta^{'}+\xi\theta=0; z=0 , \\ \theta^{'}-\xi\theta=0; z=d , \\ \varphi^{'}=0; z=0 , \\ \varphi^{'}=0; z=d . \end{aligned} \right. (S1.4)$$

Here $\xi$ is a pinning parameter, originating from the surface anisotropy, $d$ is the thickness of the magnetic film. Parameter $\xi$ may be expressed in terms of the surface anisotropy parameter $K_{s}$ as follows: $\xi=\frac{2K_{s}}{AM^{2}}$. For the further calculations we assumed $\xi d=0.5$ (the general case of partially pinned spins).

Eq. (S1.2) together with Eqs. (S1.3) and (S1.4) fully formulates the Cauchy differential equation problem. The solution of Eq. (S1.2) has the form of decaying harmonic oscillations $\theta\left( z,t \right), \varphi\left( z,t \right) \sim e^{i\left( kz-\omega t \right)-\lambda t}$. Here, assuming that $\lambda$ (which is proportional to $\alpha$) is small, the frequency $\omega$ is expressed through the wavevector $k$ as follows: $\omega^{2}=\gamma^{2}\left( H+AMk^{2} \right)\left( \tilde{H}+AMk^{2} \right)$. We should note that two values of wavevector $k$ ($k_{+}$ and $k_{-}$) correspond to the given value of the frequency $\omega$. The first one ($k_{+}$) can be either real ($k_{+}=k$) or imaginary ($k_{+}=i\chi_{+}$), depending on $\omega$, and the second one ($k_{-}$) is always imaginary ($k_{-}=i\chi$). Solutions with imaginary $k_{\pm}$ are responsible for hyperbolic (surface) terms of the modes, and can’t be neglected. The more detailed explanation was given in Ref.  [2].

Since the excited PSSW oscillations can be detected through the Faraday effect, which is sensitive to the normal component of the magnetization, we will describe the PSSWs by the $\theta$ angle. There are two types of the PSSW modes $\theta_{n}\left( z,t \right)=\theta_{n}\left( t \right)\cdot\theta_{n}\left( z \right)$:

$$\theta_{n}\left( z,t \right)=e^{-\lambda_{n}t}\sin\omega_{n}t\cdot\left\{ \begin{aligned} \cos k_{n}z^{'}+b_{n}B_{n}\cosh\chi_{n}z^{'}, n=2,4,6,\ldots\\ \sin k_{n}z^{'}+b_{n}B_{n}\sinh\chi_{n}z^{'}, n=\left( 1 \right),3,5,\ldots\end{aligned} \right. , (S1.5a)$$

$$\theta_{n}\left( z,t \right)=e^{-\lambda_{n}t}\sin\omega_{n}t\cdot\left\{ \begin{aligned} \cosh\chi_{+,n}z^{'}+\tilde{b}_{n}\tilde{B}_{n}\cosh\chi_{n}z^{'} , n=0 \\ \sinh\chi_{+,n}z^{'}+\tilde{b}_{n}\tilde{B}_{n}\sinh\chi_{n}z^{'} , n=\left( 1 \right) \end{aligned} \right. . (S1.5b)$$

Here, for simplicity, we introduced the notation: $z^{'}=z-\frac{d}{2}$ .

However, the expressions for modes $\varphi_{n}\left( z,t \right)=\varphi_{n}\left( t \right)\cdot\varphi_{n}\left( z \right)$ will also play the role for the future calculations:

$$\varphi_{n}\left( z,t \right)=e^{-\lambda_{n}t}\cos\omega_{n}t\cdot\sqrt{-b_{n}}\left\{ \begin{aligned} \cos k_{n}z^{'}+B_{n}\cosh\chi_{n}z^{'}, n=2,4,6,\ldots\\ \sin k_{n}z^{'}+B_{n}\sinh\chi_{n}z^{'}, n=\left( 1 \right),3,5,\ldots\end{aligned} \right. , (S1.6a)$$

$$\varphi_{n}\left( z,t \right)=e^{-\lambda_{n}t}\cos\omega_{n}t\cdot\sqrt{-b_{n}}\left\{ \begin{aligned} \cosh\chi_{+,n}z^{'}+\tilde{B}_{n}\cosh\chi_{n}z^{'} , n=0 \\ \sinh\chi_{+,n}z^{'}+\tilde{B}_{n}\sinh\chi_{n}z^{'} , n=\left( 1 \right) \end{aligned} \right. . (S1.6b)$$

Here, the modes symmetric with respect to the film center correspond to even *n*, the antisymmetric modes – to the odd $n$. Note, that the modes with $n=1$ may have a form of Eqs. (S1.5a)-(S1.6a) or Eqs. (S1.5b)-(S1.6b) depending on the product $\xi d$. For small product $\xi d$ the $n=1$ mode will take a form of Eqs. (S1.5a)-(S1.6a).

It should also be noted that for small values of $\xi d$ hyperbolic terms in Eqs. (S1.5a)-(S1.6a) are relatively small and these modes can be considered as «quasi-harmonic» with wavevector $k_{n}\approx\frac{\pi n}{d}-\frac{\xi}{\pi n}$ . As for the modes described by Eqs. (S1.5b)-(S1.6b), we may call them «hyperbolic» modes, as they are expressed through the sum of hyperbolic functions. The expressions for all the coefficients and wavevectors presented in Eqs.(S1.5)-(S1.6) are described in more details in Ref. [20].

А spatially non-uniform instant stimulus will excite a set of eigenmodes with different amplitudes $A_{n}$:

$$\theta\left( z,t \right)=\sum_{n=0}^{\infty} A_{n}\cdot\theta_{n}\left( z,t \right). (S1.7)$$

These amplitudes $A_{n}$ depend on the distribution of the IFE-field $h\left( z \right)$, which determines the initial conditions, and may be found using the following expression:

$$A_{n}=\frac{\gamma\Delta t}{\left( \int_{0}^{d} \varphi_{n}\left( z \right)\cdot\theta_{n}\left( z \right) dz \right)}\int_{0}^{d} h\left( z \right)\cdot\theta_{n}\left( z \right) dz . (S1.8)$$

The detailed derivation of Eq. (S1.8) is given in S3.

**S2. Optical field distribution inside the BIG layer.**

Let’s consider the case of normal incidence of light on a BIG layer. In this case, the effective field $\mathbf{H}_{IFE}=-\frac{ig}{16\pi M_{s}}\left[ \mathbf{E}\times\mathbf{E}^{*} \right]$ is obviously directly proportional to the optical field intensity: $H_{IFE}\left( z \right)\propto g\left| E\left( z \right) \right|^{2}$.

If the BIG layer is surrounded by two media, the optical field distribution has the form:

$$\begin{aligned} E\left( z \right)=A\left\{ n_{2}\cos\left( n_{2}k_{0}\left( z-d \right) \right)+in_{3}\sin\left( n_{2}k_{0}\left( z-d \right) \right) \right\},\#(S2.1) \end{aligned}$$

where

$$\begin{aligned} A=\frac{2n_{1}}{n_{2}\left( n_{1}+n_{3} \right)\cos\left( n_{2}k_{0}d \right)-i\left( n_{2}^{2}+n_{1}n_{3} \right)\sin\left( n_{2}k_{0}d \right)}.\#(S2.2) \end{aligned}$$

Here $n_{1}$ is the refractive index of the incoming medium, $n_{2}$ and $n_{3}$ are refractive indices of the BIG film and the backward medium, respectively, $i$ is the imaginary unit, $k_{0}$ is the vacuum wavelength, $z$ is the coordinate normal to the film surface, $z=0$ is at the interface between the BIG layer and the incoming medium. It can be easily shown that for the transparent media the intensity distribution has the form

$$\begin{aligned} \left| E\left( z \right) \right|^{2}=A_{0}+A_{1}\cos^{2} \left( n_{2}k_{0}\left( z-d \right)+\varphi\right),\#(S2.3) \end{aligned}$$

which corresponds to Eq. (2).

Eq. (S2.1) remains valid for the case when the backward medium is replaced by the semi-infinite photonic crystal. In this case $n_{3}$ is the complex effective refractive index of the photonic crystal defined as the relation between magnetic and electric fields of the Bloch wave at the interface:

$$\begin{aligned} n_{3}=\frac{H\left( z=0 \right)}{E\left( z=0 \right)},\#(S2.4) \end{aligned}$$

From the explicit equations for the Bloch wave fields one can obtain:

$$\begin{aligned} n_{3}=in_{4}\frac{2n_{5}\exp\left( iK\left( a+b \right) \right)-\left( n_{4}+n_{5} \right)\cos\left( k_{0}n_{4}a+k_{0}n_{5}b \right)+\left( n_{4}-n_{5} \right)\cos\left( k_{0}n_{4}a-k_{0}n_{5}b \right)}{\left( n_{4}-n_{5} \right)\sin\left( k_{0}n_{4}a-k_{0}n_{5}b \right)-\left( n_{4}+n_{5} \right)\sin\left( k_{0}n_{4}a+k_{0}n_{5}b \right)},\#(S2.5) \end{aligned}$$

where it is assumed that the elementary cell of the photonic crystal consists of two layers with refractive indices $n_{4}$ and $n_{5}$ with thicknesses $a$ and $b$, respectively. $K$ is the Bloch wavenumber for the Bloch wave propagating along the positive direction of the $z$-axis. If the optical absorption is neglected $K$ can be found from the following relation:

$$\begin{aligned} \exp\left( iK\left( a+b \right) \right)=\alpha+i\left( \mathrm{sgn} \left( \sin\left( k_{0}n_{4}a+k_{0}n_{5}b \right) \right) \right)\sqrt{1-\alpha^{2}},\#(S2.6) \end{aligned}$$

where

$$\begin{aligned} \alpha=\cos\left( k_{0}n_{4}a \right)\cos\left( k_{0}n_{5}b \right)-\frac{1}{2}\left( \frac{n_{4}}{n_{5}}+\frac{n_{5}}{n_{4}} \right)\sin\left( k_{0}n_{4}a \right)\sin\left( k_{0}n_{5}b \right),\#(S2.7) \end{aligned}$$

Eq. (S2.6) is valid for the case $\left| \alpha\right|\leq1$, which is fulfilled for the wavelengths outside the photonic bandgaps. However, the most important case is the bandgaps so let’s explore it in details. At this $\left| \alpha\right|>1$, and the Bloch wavenumber can be found from

$$\begin{aligned} \exp\left( iK\left( a+b \right) \right)=\alpha-\left( \mathrm{sgn} \alpha\right)\sqrt{\alpha^{2}-1}.\#(S2.8) \end{aligned}$$

One can see that $\exp\left( iK\left( a+b \right) \right)$ becomes real. It follows from Eq. (S2.5) that $n_{3}$ is imaginary, and Eq. (S2.1) takes the form:

$$\begin{aligned} E\left( z \right)=A\sqrt{n_{2}^{2}-n_{3}^{2}}\cos\left( n_{2}k_{0}\left( z-d \right)+\varphi\right),\#(S2.9) \end{aligned}$$

where

$$\begin{aligned} \varphi=-\mathrm{atan} \left( \frac{in_{3}}{n_{2}} \right).\#(S2.10) \end{aligned}$$

Therefore,

$$\begin{aligned} \left| E\left( z \right) \right|^{2}=\left| A \right|^{2}\left( n_{2}^{2}-n_{3}^{2} \right)\cos^{2} \left( n_{2}k_{0}\left( z-d \right)+\varphi\right),\#(S2.11) \end{aligned}$$

Eqs. (S2.10) and (S2.11) fully confirm Eqs. (3) and (4).

The phase $\varphi$ calculated by Eqs. (S2.5), (S2.7), (S2.8) and (S2.10) is shown in Fig. 3c (solid lines). For the pristine film Eqs. (S2.1), (S2.2) and (S2.3) were used. The disagreement with rigorous simulations shown by dots is caused by the fact that rigorous simulations were performed for the inclined incidence similar to the experiments. Also optical absorption was taken into account.

**S3. The deduction of formula for excitation amplitudes** $\boldsymbol{A}_{\boldsymbol{n}}$**.**

The previous formula for the excitation amplitudes $A_{n}=\frac{\beta_{n}\gamma^{2}\Delta t}{\omega_{n}d}\sum_{s} \left( H+AMk_{s}^{2} \right)\int_{0}^{d} h_{s}\left( z \right)\cdot\theta_{n}\left( z \right) dz$ , mentioned in Ref.  [2], was based on the orthogonality assumption:

$$\int_{0}^{d} \theta_{m}\left( z \right)\theta_{n}(z)dz\approx\frac{d}{\beta_{n}}\delta_{mn} \left( \beta_{n=0}=1, \beta_{n\neq0}=2 \right) . (S3.1)$$

However, this is valid only for not large values of the pinning parameter ( $\xi d<1.5-2.0$ ). We would like to have the more complete theory, which will be valid for any values of the pinning parameter. In this case, we should replace Eq. (S3.1) by the following:

$$\int_{0}^{d} {\varphi_{m}\left( z \right)\theta}_{n}\left( z \right)dz=\frac{d}{\beta_{n}^{'}}\delta_{mn} . (S3.2)$$

Here the constants $\beta_{n}^{'}$ may be found precisely, as will be shown later. Eq. (S3.2) represents an analogue of orthogonality condition, but one more basis $\varphi_{n}$ (biorthogonal basis) is used to satisfy the condition, because $\theta_{n}$ functions are not orthogonal to each other in a general case. We will prove the validity of the Eq. (S3.2) below.

Let us write the linearized LLG-equations (Eq. (S3.2)) for the separate modes $\theta_{m}\left( z,t \right)=\theta_{m}\left( t \right)\cdot\theta_{m}\left( z \right)$ and $\varphi_{m}\left( z,t \right)=\varphi_{m}\left( t \right)\cdot\varphi_{m}\left( z \right)$. Neglecting the damping and considering that $\theta_{m}\left( t \right)\sim\sin\omega_{m}t$ , $\varphi_{m}\left( t \right)\sim\cos\omega_{m}t$, we will obtain:

$$\left\{ \begin{aligned} \omega_{m}\theta_{m}\left( z \right)=\gamma H\varphi_{m}\left( z \right)-\gamma AM\varphi_{m}^{''}\left( z \right) , \\ \omega_{m}\varphi_{m}\left( z \right)=\gamma\tilde{H}\theta_{m}\left( z \right)-\gamma AM\theta_{m}^{''}\left( z \right) . \end{aligned} \right.(S3.3)$$

We will multiply the first equation in (S3.3) by $\varphi_{n}\left( z \right)$ and the second – by $\theta_{n}\left( z \right)$. Then, integrating them and introducing the notations like $\int_{0}^{d} \varphi_{m}\left( z \right)\theta_{n}\left( z \right)dz=(\varphi_{m} \theta_{n})$ , we will have:

$$\left\{ \begin{aligned} \omega_{m}\left( \theta_{m} \varphi_{n} \right)=\gamma H{(\varphi}_{m} \varphi_{n})-\gamma AM\left( \varphi_{m}^{''} \varphi_{n} \right) , \\ \omega_{m}\left( \varphi_{m} \theta_{n} \right)=\gamma\tilde{H}\left( \theta_{m} \theta_{n} \right)-\gamma AM\left( \theta_{m}^{''} \theta_{n} \right). \end{aligned} \right.(S3.4)$$

We can transform the last terms in equations (S3.4) with the use of double integration by parts:

$$\left\{ \begin{aligned} \left( \varphi_{m}^{''} \varphi_{n} \right)=\left( \varphi_{m}^{'}\varphi_{n}-\varphi_{m}\varphi_{n}^{'} \right)\left| {d \atop0} \right.+\left( \varphi_{n}^{''} \varphi_{m} \right) , \\ \left( \theta_{m}^{''} \theta_{n} \right)=\left( \theta_{m}^{'}\theta_{n}-\theta_{m}\theta_{n}^{'} \right)\left| {d \atop0} \right.+\left( \theta_{n}^{''} \theta_{m} \right) . \end{aligned} \right. (S3.5)$$

Using the boundary condition (S1.4), we will get from Eq. (S3.5): $\left( \varphi_{m}^{''} \varphi_{n} \right)=\left( \varphi_{n}^{''} \varphi_{m} \right)$ , $\left( \theta_{m}^{''} \theta_{n} \right)=\left( \theta_{n}^{''} \theta_{m} \right)$. Taking into account that ${(\varphi}_{m} \varphi_{n})={(\varphi}_{n} \varphi_{m})$ , $\left( \theta_{m} \theta_{n} \right)=(\theta_{n} \theta_{m})$, we see that the right part of the equations (S3.4) is invariant under the permutation of indices $m,n$ , which, thus, also leads to the invariance of the left part of the equations (S3.4):

$$\left\{ \begin{aligned} \omega_{m}\left( \theta_{m} \varphi_{n} \right)=\omega_{n}\left( \theta_{n} \varphi_{m} \right) , \\ \omega_{m}\left( \varphi_{m} \theta_{n} \right)=\omega_{n}\left( \varphi_{n} \theta_{m} \right). \end{aligned} \right.(S3.6)$$

$$\left\{ \begin{aligned} \left( \theta_{m} \varphi_{n} \right)=\frac{\omega_{n}}{\omega_{m}}\left( \theta_{n} \varphi_{m} \right) , \\ \left( \theta_{n} \varphi_{m} \right)=\frac{\omega_{n}}{\omega_{m}}\left( \theta_{m} \varphi_{n} \right). \end{aligned} \right.(S3.7)$$

Substituting the second equation into the first in (S3.7), we will finally have:

$$\left( \theta_{m} \varphi_{n} \right)=\frac{{\omega_{n}}^{2}}{{\omega_{m}}^{2}}\left( \theta_{m} \varphi_{n} \right) . (S3.8)$$

From Eq. (S3.8) it follows that in a case when $\frac{\omega_{n}}{\omega_{m}}\neq1$ (which is valid, when $m\neq n$), the term $\left( \theta_{m} \varphi_{n} \right)$ is equal to zero. This means, that functions $\varphi_{n}$ constitute a biorthogonal basis to functions $\theta_{n}$ : $\left( \theta_{m} \varphi_{n} \right)\sim\delta_{mn}$ , which proves the validity of the Eq. (S3.2). Using this fact, we can deduce the more precise formula for the excitation amplitudes of different PSSW modes.

For obtaining the expression for the excitation amplitudes we can use the initial condition on $\dot{\theta}$:

$$\dot{\theta}\left( z,t=\Delta t \right)=\sum_{n=0}^{\infty} A_{n}\omega_{n}\theta_{n}\left( z \right)=\gamma^{2}\Delta t\left[ H\cdot h\left( z \right)-AM\cdot h^{''}\left( z \right) \right]. (S3.9)$$

In a real experiment the IFE-field may be proportional to the superposition of the harmonic functions $h_{s}\left( z \right)\sim\sin k_{s}z, \cos k_{s}z$ (including $k_{s}=0$): $h\left( z \right)=\sum_{s} h_{s}\left( z \right)$. In this case, the Eq. (S3.9) may be simplified using ${h_{s}}^{''}\left( z \right)=-k_{s}^{2}\cdot h_{s}\left( z \right)$ :

$$\sum_{n=0}^{\infty} A_{n}\omega_{n}\theta_{n}\left( z \right)=\gamma^{2}\Delta t\sum_{s} \left[ H+AMk_{s}^{2} \right]\cdot h_{s}\left( z \right). (S3.10)$$

Now, we shall use the previously obtained “biorthogonal” condition (Eq. S3.2). Multiplying Eq. (S3.10) by $\varphi_{m}\left( z \right)$ and integrating it by $z$ with the further change of index $m$ by $n$, we will obtain the expression for the amplitudes of modes $A_{n}$:

$$A_{n}=\frac{\beta_{n}^{'}\gamma^{2}\Delta t}{\omega_{n}d}\sum_{s} \left( H+AMk_{s}^{2} \right)\int_{0}^{d} h_{s}\left( z \right)\cdot\varphi_{n}\left( z \right) dz. (S3.11)$$

Here $\beta_{n}^{'}=\frac{d}{(\theta_{n} \varphi_{n})}$ . If we express the frequency $\omega_{n}$ through the wavevector $k$, we will get exactly the form of the equations presented in Ref.  [2], while the only difference will be in changing the function $\theta_{n}\left( z \right)$ in the expression for the amplitudes by the function $\varphi_{n}\left( z \right)$.

We shall note, that in a limiting case $\xi d\to0$, the functions $\varphi_{n}$ and $\theta_{n}$ take the same form up to the normalizing coefficient $\sqrt{-b_{n}}$. That is the reason, why the previous approach for calculating the excitation amplitudes was still applicable for the small values of $\xi d$ product.

However, there is one more, even more simple way to obtain the expression for the excitation amplitudes of PSSWs. Let us now use the initial condition (Eq. (S1.3)) on $\varphi$:

$$\varphi\left( z,t=\Delta t \right)=\sum_{n=0}^{\infty} A_{n}\varphi_{n}=\gamma\Delta t\cdot h\left( z \right) . (S3.12)$$

Here we used the fact, that the modes $\varphi_{n}$ and $\theta_{n}$ are not independent and, thus, have the same amplitudes $A_{n}$ . In order to find these amplitudes, we should multiply Eq. (S3.12) by $\theta_{m}\left( z \right)$ (but not by $\varphi_{m}\left( z \right)$, as was shown before) and integrate it by $z$ with the further change of index $m$ by $n$. With the help of the previously obtained “biorthogonal” condition (Eq. (S3.2)) we will get the expression for the amplitudes of modes $A_{n}$ (which coincides with the aforementioned Eq.(S1.8)):

$$A_{n}=\frac{\gamma\Delta t}{(\varphi_{n} \theta_{n})}\int_{0}^{d} h\left( z \right)\cdot\theta_{n}\left( z \right) dz=\gamma\Delta t\frac{(\theta_{n} h)}{(\theta_{n} \varphi_{n})} . (S3.13)$$

Here $\left( \varphi_{n} \theta_{n} \right)=\int_{0}^{d} \varphi_{n}\left( z \right)\cdot\theta_{n}\left( z \right) dz$ and can be estimated for small values of $\xi d$ as: $\left( \varphi_{n} \theta_{n} \right)=\frac{\sqrt{-b_{n}}}{\beta_{n}}d=\frac{d}{\beta_{n}}\sqrt{\frac{\tilde{H}+AMk_{n}^{2}}{H+AMk_{n}^{2}}}, (\beta_{n=0}\approx1, \beta_{n\neq0}\approx2, k_{n=0}^{2}=-\chi_{n=0}^{2})$ .

**S4. The inverse Faraday effect effective magnetic field distribution for MPhS with 125 nm thick BIG layer.**

To selectively excite the 1st and the 2nd modes the thickness of the BIG layer should be made 125 nm. Calculated transmission and Faraday angle spectra for this case (the Bragg mirror is similar to the one presented in the manuscript, i.e. [SiO_2_(105 nm)/TiO_2_(66 nm)]x4) are presented in Figure S1a. The transmission spectrum contains a similar bandgap as for the original structure. However, the “defect” mode is now shifted to ~5 nm (a feature in the Faraday angle spectra). The distribution of the IFE field is shown in Figure S1b. As the wavelength increases, the number of nodes increases. For 500 nm there are 3 nodes, which can result in the 3rd order spin-wave mode excitation. Ultimately, at 600 nm the second order mode can be excited and the first order mode is at 700 nm.

| 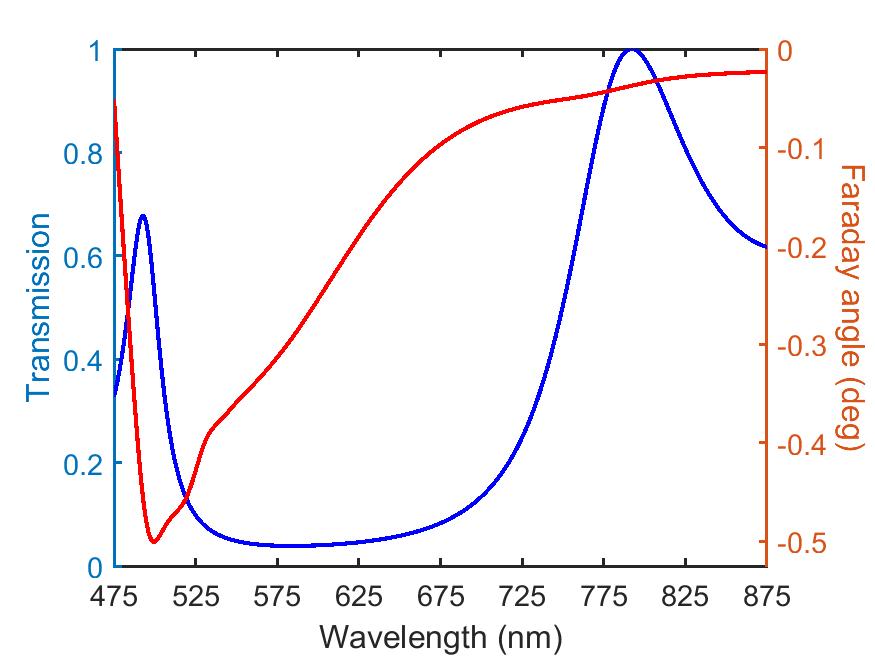  a | 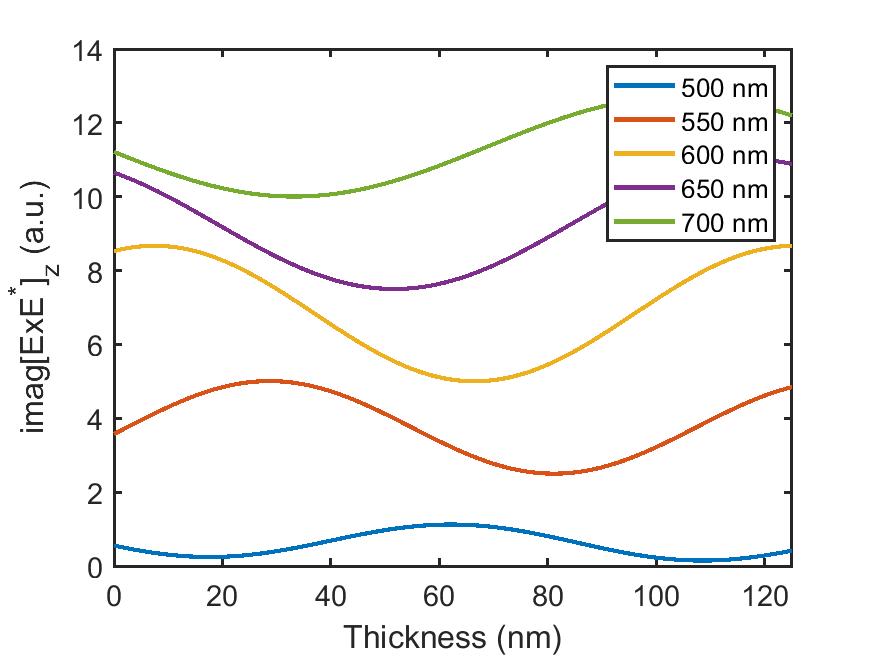  b |
| --- | --- |

Figure S1. Transmission and Faraday rotation spectra of the MPhS with 125 nm thick BIG layer (a) and distribution of the IFE effective magnetic field within BIG layer at different wavelength.

**References:**

1. A. G. Gurevich and G. A. Melkov, *Magnetization Oscillations and Waves* (CRC Press, 2020).

2. V. A. Ozerov, D. A. Sylgacheva, M. A. Kozhaev, T. Mikhailova, V. N. Berzhansky, M. Hamidi, A. K. Zvezdin, and V. I. Belotelov, "One-dimensional optomagnonic microcavities for selective excitation of perpendicular standing spin waves," J Magn Magn Mater **543**, 168167 (2022).
